# CodABC: A Computational Framework to Coestimate Recombination, Substitution, and Molecular Adaptation Rates by Approximate Bayesian Computation

**DOI:** 10.1093/molbev/msu411

**Published:** 2015-01-09

**Authors:** Miguel Arenas, Joao S. Lopes, Mark A. Beaumont, David Posada

**Affiliations:** ^1^Centre for Molecular Biology “Severo Ochoa,” Consejo Superior de Investigaciones Científicas (CSIC), Madrid, Spain; ^2^Departamento de Bioquímica, Genética e Inmunología, Universidad de Vigo, Vigo, Spain; ^3^Instituto Gulbenkian de Ciencia, Oeiras, Portugal; ^4^School of Mathematical Sciences and School of Biological Sciences, University of Bristol, University Walk, Bristol, United Kingdom

**Keywords:** approximate Bayesian computation, recombination, molecular adaptation, substitution rate, coding data

## Abstract

The estimation of substitution and recombination rates can provide important insights into the molecular evolution of protein-coding sequences. Here, we present a new computational framework, called “CodABC,” to jointly estimate recombination, substitution and synonymous and nonsynonymous rates from coding data. CodABC uses approximate Bayesian computation with and without regression adjustment and implements a variety of codon models, intracodon recombination, and longitudinal sampling. CodABC can provide accurate joint parameter estimates from recombining coding sequences, often outperforming maximum-likelihood methods based on more approximate models. In addition, CodABC allows for the inclusion of several nuisance parameters such as those representing codon frequencies, transition matrices, heterogeneity across sites or invariable sites. CodABC is freely available from http://code.google.com/p/codabc/, includes a GUI, extensive documentation and ready-to-use examples, and can run in parallel on multicore machines.

Understanding adaptation is one of the central questions in evolutionary biology (e.g., Nielsen 2005; Barrick et al. 2009; Jones et al. 2012). At the molecular level, the estimation of nonsynonymous/synonymous rate ratio (ω) has played a fundamental role in the identification of loci and codon sites under selective pressure (i.e., Yang and Nielsen 2000; Perez-Losada et al. 2009; Yang et al. 2009). However, the estimation from real data of this parameter is not trivial, and other evolutionary processes such as recombination can introduce a bias (Anisimova et al. 2003; Shriner et al. 2003; Arenas and Posada 2010). As a consequence, there is a need for methods of inference that can allow for different evolutionary scenarios in which multiple parameters are jointly estimated. Indeed, for such complex models it can be impossible to derive analytical formulae, or the likelihood function may be computationally too expensive to evaluate. In such cases, an approximate Bayesian computation (ABC) approach (Beaumont 2010; Csillery et al. 2010) can provide a reasonable solution. We have recently proposed an ABC strategy for the joint estimation of recombination, nonsynonymous/synonymous rate ratios, and substitution rates that outperforms other methods based on maximum likelihood and that is quite robust to model misspecification (Lopes et al. 2014). Here, we present a user-friendly computational tool that implements this methodology, called “CodABC.” In contrast to other ABC tools, CodABC allows for the analysis of coding data while jointly considering multiple parameters and complex codon substitution models. As with any ABC method, CodABC uses summary statistics designed to extract evolutionary information from coding data. Moreover, CodABC is able to perform ABC under both multiple rejection and regression strategies.

## New Approaches: CodABC

An analysis with CodABC consists of three main steps: Simulation of coding data, computation of summary statistics and joint estimation of recombination, ω, and codon substitution rates.

1. The simulation of coding data is performed with the coalescent simulator *CoalEvol* (Arenas and Posada 2014), which implements different evolutionary scenarios with recombination (including intracodon breakpoints), haploid/diploid data and longitudinal sampling. Coding sequences are evolved along the simulated genealogies under the GY94 codon model (Goldman and Yang 1994), combined with any typical 4 × 4 nucleotide substitution model (e.g., Pond and Muse 2005; Anisimova and Kosiol 2009), accommodating rate variation among sites and a proportion of invariable sites (Yang 1994). This simulation can be parameterized according to user-specified prior distributions (see Arenas and Posada 2014).
2. A total of 26 summary statistics are computed to encapsulate the information in the observed and simulated data. These summary statistics consist of three fast recombination tests (pairwise homoplasy index [Bruen et al. 2006], neighbor similarity score [Jakobsen and Easteal 1996], and maximum chi-squared [Maynard Smith 1992]); the mean, standard deviation, skewness and kurtosis of diversity and heterozygosity at codon and amino acids levels, the number of segregating sites at nucleotide, codon and amino acid levels, and a series of summary statistics that simultaneously consider diversity at the codon and amino acid levels. We have previously shown that this set of summary statistics is able to extract a substantial amount of the evolutionary information of interest from coding alignments (Lopes et al. 2014).
3. In the last step, CodABC estimates the three parameters of interest using the *abc* R package (Csillery et al. 2012): 1) Scaled recombination rate *ρ* = 4*Nrl*, where *N* is the effective population size, *r* is the recombination rate per nucleotide, and *l* is the number of nucleotides in the alignment; 2) nonsynonymous/synonymous rate ratio *ω*; and 3) scaled codon substitution rate *θ* = 4 *NµL*, where *µ* is the substitution rate per codon and *L* is the number of codons in the alignment. Note that other parameters that are used for simulating data during the ABC procedure are treated as nuisance parameters—sampled according to a prior distribution but not estimated—such as codon frequencies, substitution rates among nucleotides, rate variation among sites or proportion of invariable sites, which allow distinct evolutionary scenarios to be explored. The estimation step can be carried out under a rejection or a weighted multiple linear regression approach (Beaumont et al. 2002; Blum and François 2010; Csillery et al. 2010).

The user of CodABC can specify the number of simulations to consider, the tolerance level, different transformations of the data (none, log, or logit), corrections for heteroscedasticity, and the subset of the summary statistics that will be used for the estimation. Detailed recommendations are described in the software documentation, but see also CodABC Validation section. In general, we found that 50,000 simulations can be a good starting point, but different data sets may require a larger number of simulations depending on the amount of information (e.g., small data sets may require more simulations).

Conveniently, CodABC includes a user-friendly GUI for an easy parameterization of the whole estimation procedure. Because the simulation of coding data is commonly much slower than the simulation of nucleotide or amino acid data, CodABC can run the simulations and the computation of the summary statistics in parallel on multicore machines, allowing for a significant reduction of the computation time (see below). CodABC is a pipeline written in Java, C, Perl, and R, freely available from http://code.google.com/p/codabc/. The package includes executables, source code, detailed documentation, and example input files.

## CodABC Validation

**Fig. 1.**
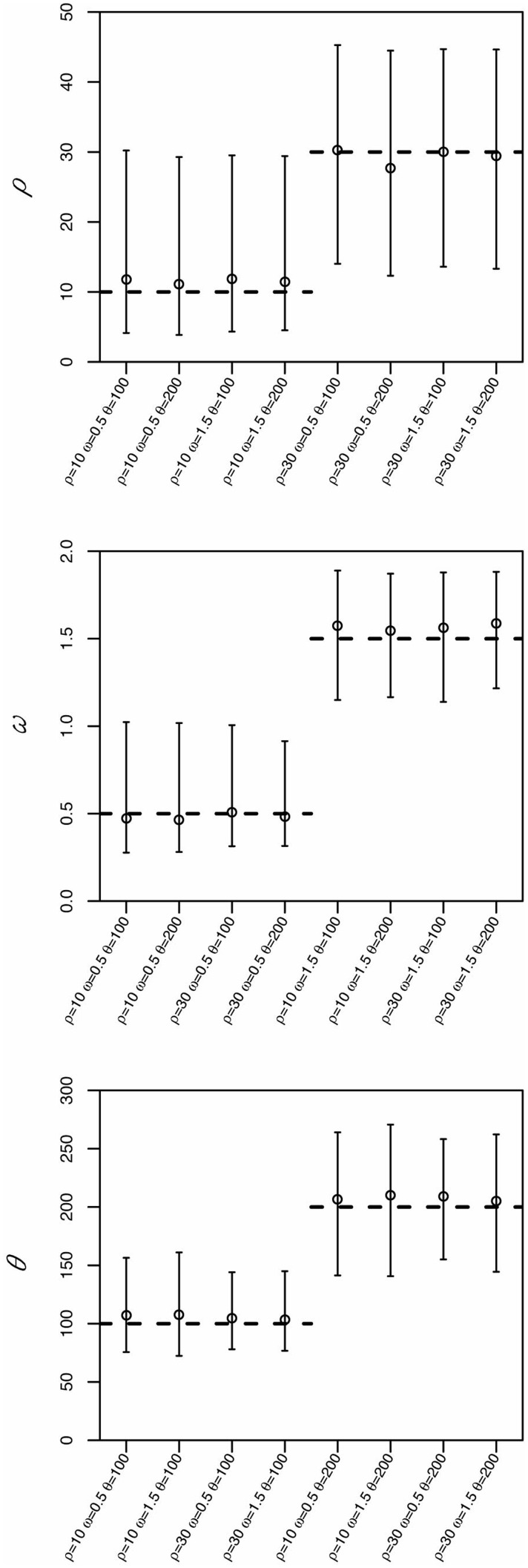
Accuracy of CodABC using simulated data. For each combination of ρ, θ, and ω, we present the corresponding estimates for ρ (top), ω (middle), and θ (down). Dashed lines indicate the true value. Points present the mode of the prior distributions and error bars indicate the 95% CI.

We have previously shown that ABC can generate more accurate estimates than maximum-likelihood methods under a number of scenarios (Lopes et al. 2014). Here, and in order to benchmark and validate the specific CodABC implementation, we carried a new simulation study. We simulated coding sequences under different values of *ρ* (10 and 30), *ω* (0.5 and 1.5) and *θ* (100 and 200), for alignments of 15 sequences with 300 codons, assuming a fixed effective population size of 1,000 individuals, and a GY94 codon model (Goldman and Yang 1994) with a transition/transversion rate ratio of 0.5. For every combination of parameters (2 × 2 × 2 = 8 combinations), we simulated 100 alignments. For each data set, we used CodABC to obtain estimates of *ρ*, *ω*, *θ*, with a total of 50,000 simulations parameterized under the following wide prior distributions: *ρ* = Uniform(0,50), *θ* = Uniform(0,300), and *ω* = Uniform(0,2), which encompass values that are commonly observed in real data (e.g., Stumpf and McVean 2003; Carvajal-Rodriguez et al. 2006; Perez-Losada et al. 2009). ABC estimates were obtained assuming an acceptance rate of 0.2%, giving 100 points, adjusted with a weighted multiple linear regression on logit-transformed values, as in Lopes et al. (2014). The parameter estimates obtained were generally accurate and in good agreement with previous tests (Lopes et al. 2014), validating the CodABC implementation (fig. 1).

**Fig. 2.**
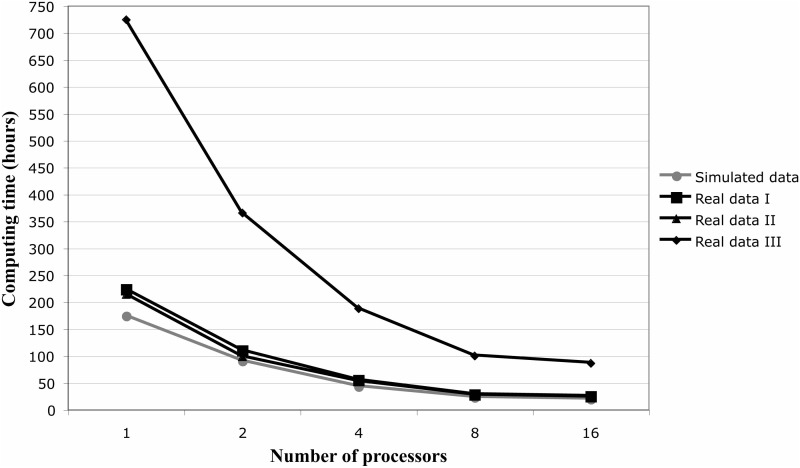
CodABC computing times. The simulated data contain 15 sequences with 900 nucleotides. The first real data set contains 22 sequences with 864 nucleotides. The second real data set contains 20 sequences with 894 nucleotides. The third real data set is the biggest and contains 55 sequences with 1,449 nucleotides. Prior distributions: ρ: U(0,50), θ: U(0,300), and ω: U(0,2). The analyses were run on an Intel Xeon CPU 2.33 GHz with 24 cores.

In order to provide an idea of typical running times, we also reanalyzed with CodABC three HIV-1 data sets, including two already studied in Lopes et al. (2014). HIV-1 is particularly interesting to analyze due to the very high recombination and substitution rates (Mansky and Temin 1995; Robertson et al. 1995), and its evolution under strong selective pressures promoted by the immune system and antiretroviral therapy (e.g., Poon et al. 2007). The first data set included 22 sequences and 288 codons—intrapatient dynamics under antiretroviral therapy—(Malet et al. 2009), the second included 20 sequences and 298 codons—gp41 sequences of type 1 subtype C from India—(Agnihotri et al. 2006), and the third data set is the biggest and included 55 sequences and 483 codons—a genetic characterization of a new circulating recombinant form in China—(Zeng et al. 2012). We ran a total of 50,000 simulations under the same prior distributions used for the analysis of the simulated data above. The analyses of these data sets took 7 days for the smallest data set and 30 days for the biggest on a single core, but the running times were drastically reduced when using four (43 and 188 h for the smallest and biggest data sets, respectively) or eight cores (22 and 99 h for the smallest and biggest data sets, respectively) (Intel Xeon CPU 2.33 GHz) (fig. 2). As expected, bigger data sets, with more and longer sequences, lead to longer computer times and thus we recommend running them in parallel on multicore machines. Indeed, we note that high recombination rates in the simulation prior might result in large ancestral recombination graphs that imply larger simulation times (Arenas and Posada 2012).

## Discussion

We have introduced a new ABC tool for the estimation of nonsynonymous/synonymous rate ratio, recombination and codon substitution rates from coding sequence alignments. Key aspects of CodABC are the implementation of coalescent simulations under a variety of models of evolution, the consideration of flexible prior distributions and the joint estimation of different evolutionary parameters. Many of these features are commonly unavailable in other analytical methods (e.g., those based on maximum-likelihood approaches [see Li and Stephens 2003; Wilson and McVean 2006]). We have shown that with a reasonable computational effort CodABC can be quite accurate, often more than maximum-likelihood methods based on more approximate models (Lopes et al. 2014). Nevertheless, some care should be taken when specifying the ABC procedure, for example the number of simulations or the acceptance rate. We recommend the use of the GUI to define the entire analysis, as this tool checks for potential setting errors. As a starting point, we recommend to perform 50,000 simulations and to consider an acceptance rate not lower than 0.2% for simulated data for which we know the model of evolution, and as much as 500,000 simulations and an acceptance rate of at least 1,000 data sets for real data. The prior distributions should be carefully defined, making sure that the values of the parameters are biologically reasonable, and that the value of the summary statistics for the simulated data and the data set under study are similar. It is also important to obtain a good coverage of the space of the parameters through extensive simulations. Repeating the analysis with an increasing number of simulations, and different acceptance rates, can help in identifying the number of simulations required for obtaining reliable estimates in a particular analysis.
